# Influence of an Aquatic Therapy Program on Perceived Pain, Stress, and Quality of Life in Chronic Stroke Patients: A Randomized Trial

**DOI:** 10.3390/ijerph17134796

**Published:** 2020-07-03

**Authors:** Sagrario Pérez-de la Cruz

**Affiliations:** Department of Nursing, Physiotherapy and Medicine, University of Almería, La Cañada de San Urbano, 04120 Almería, Spain; spd205@ual.es; Tel.: +34-9-50214574

**Keywords:** stroke, Ai Chi, aquatic therapy, pain, quality of life

## Abstract

Pain and depressive states may have a negative impact on the quality of life of individuals with stroke. The aim of this study was to evaluate the effects of a program of Ai Chi aquatic therapy on pain, depression, and quality of life in a sample of people with stroke. Forty-five participants received physiotherapy treatment on dry land (control group), an experimental group received aquatic Ai Chi therapy, and a combined therapy group received alternating sessions of physiotherapy on dry land and aquatic Ai Chi therapy. The Visual Analog Scale (VAS) scale for pain, the resilience scale, and the SF-36 quality of life scale were used as outcome measures. Statistically significant differences were found in the experimental group and the combined intervention group for post treatment pain and resilience (*p* < 0.001). Concerning the SF-36, statistically significant changes (*p* < 0.01) were found in the experimental group and the combined therapy group for all items except general health, vitality, and social function, where no between group differences were observed (*p* = 0.001). In conclusion, physical exercise performed in water has positive effects on several factors that contribute towards improving the mood and quality of life of people with acquired brain injury.

## 1. Introduction 

Stroke is a neurological disease caused by the obstruction of normal blood flow due to vessel rupture or blockage, causing damage to brain tissue [1]. Worldwide, stroke is the second leading cause of death [2] and the third most common cause of disability [3], representing the first cause of severe disability of neurological origin in adults [4]. In addition, fifty percent of ischemic stroke survivors have a permanent disability.

Alongside the motor and physical disorders, sensory, language, and perceptual deficits, together with impaired recognition, are typical symptoms of the disease, depending on the location of the lesion [1,5]. Furthermore, post-stroke depression (PSD) is the most frequent neuropsychiatric complication after a stroke [6,7,8]. In addition, many other neuropsychiatric symptoms may appear, such as anxiety, irritability, agitation, emotional incontinence, sleep disturbances, behavioral disorders such as disinhibition, apathy, fatigue, and psychotic symptoms, including delusion and hallucinations [7,8]. Neuropsychiatric disorder is the main factor for poor prognosis in functional recovery after a stroke, often associated with worse functional and cognitive recovery, a greater decline in activities of daily living, social and interpersonal life, poorer quality of life, and higher mortality (up to 10 times higher compared to subjects without stroke) [9].

Although the prevalence of PSD is high, it is often under-diagnosed and under-treated. Systematic reviews published to date show an overall prevalence of 33%, while other studies reveal that this can vary between 25% and 79% [10,11,12]. According to a review by Carod-Artal, in the first two weeks, the prevalence of PSD ranges from 6% to 40%, at three months it reaches 50%, and after a year it ranges from 20% to 50%, decreasing to 20% at two years [7]. Although there are discrepancies in the definition of this disorder, approximately one in every three patients are expected to develop PSD.

Numerous risk factors are associated with the appearance of PSD, such as more severe motor deficits, greater disability, or more limited social support. These factors must be identified, to enable the application of early prevention and treatment strategies [7]. At present, there is insufficient evidence to establish a relationship between the lateralization of stroke and the risk of presenting PSD.

Despite its detrimental effects on recovery and quality of life, there is no reliable and universal treatment for PSD. Historically, most antidepressant therapies influence the serotonergic, adrenergic, and/or dopaminergic systems with the aim of increasing the synaptic availability of serotonin, noradrenaline, and dopamine [8,13]. Another factor that can significantly contribute to increased levels of depression is decreased physical activity [14]. Regulating physical exercise positively alters symptoms of depression, thus promoting mental health [15]. In addition, exercise facilitates and encourages social interactions with other people. Viable and effective interventions can provide both physical and mental health benefits to these individuals [14,16]. Hence, one of the options to address this condition is to consider the environment where the physical activity takes place. For most patients, the aquatic environment is a distracting and enjoyable environment, which may further support their ability to cope with the situation.

Ai Chi is a novel aquatic therapy that combines the concepts of Tai Chi with conventional water therapy techniques. This form of aquatic exercise involves a total of 19 standardized movement patterns that emphasize the coordination of body movements with breathing and specific patterns [17]. Ai Chi is safe, standardized, does not require equipment, and therefore enables self-regulated practice. Use of these programs should be promoted if they are proven to yield beneficial results for patients with mental health impairments due to neurological disorders. Ai Chi addresses the need for an aquatic exercise intervention that can be taught in a group setting, while also allowing individuals to continue to practice the exercises on their own if they so desire [18].

Therefore, the aim of this study was to evaluate the effects of twelve weeks of treatment receiving Ai Chi aquatic therapy sessions, dry therapy, or combined therapy (aquatic therapy and dry therapy) on pain, depression, and quality of life among people diagnosed with stroke.

## 2. Materials and Methods 

### 2.1. Design

This study was a single-blind, randomized controlled trial (NCT04168164).

### 2.2. Participants

This study was conducted with individuals diagnosed with chronic stroke attending three associations for people with acquired brain injury in Spain, between February and September 2018.

The following inclusion criteria were applied: (1) subjects with stroke occurring at least one year before the start of therapy; (2) patients who were able to move at least 10 m with the help of an assistive device or another person; (3) the ability to tolerate interventions and assessments; and (4) the ability to follow verbal commands. The exclusion criteria were: (1) history of previous stroke or other adjuvant and/or degenerative neurological diseases; (2) history of cardiovascular disorders, such as heart failure or arrhythmia; (3) cognitive impairment identified using the Mini-Mental State Examination (MMSE) <24; and (4) the inability to follow verbal instructions. 

All participants initially declared eligible to participate in this program, provided their consent after being informed of the study. This study was conducted in accordance with the regulatory standards of good clinical practice and the Helsinki Declaration (2013) and approved by the Bioethics Committee of the University of Almería (UALBIO2017/008).

The 41 patients who met the criteria were randomly divided into one of three groups (control group, experimental group, and combined group) using random numbers in sealed envelopes (simple random sampling, using the SPSS statistical program). These numbers were generated by random number tables. 

**Figure 1:**
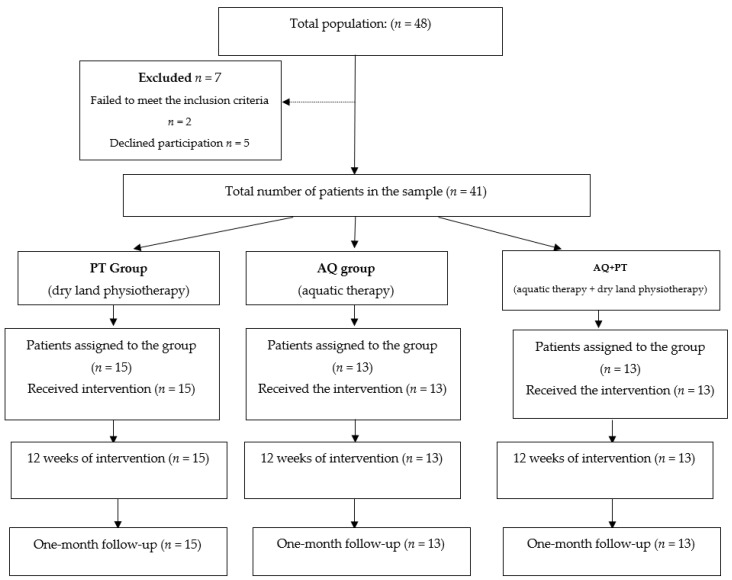
Participant selection process.

The selection process of study participants is shown below, in Figure 1. 

### 2.3. Outcome Measures

#### 2.3.1. The Visual Analog Scale (VAS)

The VAS is a continuous, single-item scale for evaluating pain intensity. This scale is based on a 10 cm line with endpoints labelled “no pain” on the left and “worst pain” on the right. The pain intensity ranges from 0 to 10, where 0 = no pain and 10 = the worst possible pain. Patients were asked to mark the place on the VAS scale corresponding to their pain level. This scale is a valid and common tool for measuring pain intensity [19].

#### 2.3.2. Resilience

Resilience is the ability to prevail, grow, be strong, and succeed in the face of adversity. It is a characteristic of people who, despite being born and living in high-risk situations, develop to become psychologically healthy and successful. This scale is based on the Connor-Davidson Resilience Scale (CD-RISC10 ©) [20]. The Spanish version of the same has been used in this study [21]. This instrument consists of 10 self-report items, each rated on a Likert type scale from 0 (not true) to 4 (true almost all the time). In the original scale, the 10 items are loaded in one dimension only. The total score ranges from 0 to 40, with higher scores indicating greater resilience. This questionnaire showed good psychometric properties in the validation study conducted in the US population (Cronbach’s alpha of 0.89).

#### 2.3.3. SF-36

We used the validated Spanish version of the SF-36 questionnaire [22]. The SF-36 is an assessment tool for evaluating quality of life which is both easy-to-administer and understandable. The psychometric properties of the SF-36 questionnaire have been widely studied, with proven reliability, validity, and sensitivity, both for the original version and the Spanish version, for the general population, and patients with different disorders. This multidimensional questionnaire evaluates the positive and negative aspects of health. It consists of 36 items comprising eight domains: functional capacity (10 items), physical aspects (four items), pain (two items), general health (five items), vitality (four items), social aspects (two items), emotional aspects (three items), mental health (five items), and a comparative assessment question between current health conditions and those from one year ago. Scores range from zero to 100, with zero being the worst result and 100 the best. This score is calculated by domain.

### 2.4. Procedure

All participants signed an informed consent form before participating in the study and during a study presentation briefing. The principal investigator personally visited the participating associations to describe the study in detail. During these sessions, the study schedule was explained, along with recommendations regarding appropriate clothing for participation in the activities.

Evaluations were conducted by a single evaluator at the beginning of the study and after the treatment period. Finally, subjects were re-evaluated at a follow-up four weeks after completing the program. The evaluator was blinded to avoid bias.

None of the participants experienced any significant treatment-related adverse events, and all patients in the intervention groups were fully compliant with the intervention program. There were no dropouts. All the sessions performed in the water were taught by an external physiotherapist, over a 12-week period. The sessions lasted 45 min and were conducted twice weekly. The participants performed the exercise in a therapeutic pool 20 m long × 6 m wide, with a water depth of 1.4 m. The water temperature was 34 to 36 °C with an air temperature of 24 °C. Another physiotherapist led the dry land therapy with the same number of sessions and durations.

### 2.5. Intervention

#### 2.5.1. Dry Land Physiotherapy (Control Group)

The 15 participants in the dry land therapy group received two sessions of physiotherapy per week for 12 weeks (a total of 24 sessions). Each session was 45 min long and consisted of an initial warm-up, lasting 10 min. At this stage, participants performed walking exercises, trunk mobility and active mobilization exercises for the upper and lower limbs. The central part of the session consisted of aerobic activities and strength and coordination exercises, with a total duration of approximately 30 min, to conclude with activities of daily living, balance work, proprioception, muscle relaxation, and stretching. For this purpose, different positions were alternated during the proposed activities, such as standing, sitting, and supine, while also including balance activities. Subsequently, more advanced postures and transitions were progressively incorporated.

#### 2.5.2. Aquatic Ai Chi (Experimental Group)

The 13 patients assigned to the aquatic therapy group (experimental group) received the same number of sessions as the members of the dry therapy group, with the same session length (45 min). The intervention was performed by an expert physiotherapist trained in clinical Ai Chi. The type of movement required to correctly perform this therapy is slow and continuous, participants must be mindful of body alignment, accompanying their movements with deep diaphragmatic breathing and a state of relaxation. The mental focus is produced by the fluid movement, proper body alignment, and coordinated breathing. 

Ai Chi consists of 19 movements or katas, performed in coordination with a constant breathing pattern of approximately 14−16 breaths per minute. The first six movements/katas are based on movements which are similar to Qigong, with a relatively static and symmetrical body posture. The progression is characterized by a gradual increase in difficulty, from static to dynamic, from symmetric movements to rotations and asymmetric gestures, and from visual to non-visual control (activation of the vestibular system). Relaxation is induced by the slow and ample movements of the arms and legs and by the focus on breathing.

#### 2.5.3. Combined Therapy Group

This group (*n* = 13) received both aquatic and dry land therapy sessions. Thus, dry land physiotherapy and Ai Chi aquatic therapy sessions were alternated in the same conditions as the participants in the control and experimental groups. Therefore, this group received the sum of the therapies of the other two groups. 

### 2.6. Stastistical Analysis

Descriptive statistics are reported as the mean (SD). The normality of the distribution of all variables was assessed using the Shapiro–Wilk statistical test. The effect of the three different rehabilitation protocols was assessed by a two-factor analysis of variance: the first factor was the effectiveness of each of the therapies, and the second factor was the duration of the effect of the therapy employed in each of the intervention groups.

To determine whether a treatment improves study variable scores compared to another, two-factor ANOVA tests were performed with repeated measures, using the general linear model (GLM) procedure. A value of *p* < 0.05 was considered statistically significant. When multiple comparisons were made, a Bonferroni correction was applied. All analyses were performed using the SSPS-23 statistical package.

## 3. Results

Forty-one individuals diagnosed with stroke were recruited for the study. Of these, fifteen individuals received physiotherapy on dry land (mean age = 62.7 years and SD = 13.4), including 53.3% women (8 subjects) and 46.6% men (7 subjects). Thirteen individuals received aquatic therapy (6 women and 7 men, mean age = 63.8 years and SD = 13.6); and finally, 13 patients received both aquatic therapy and dry land physiotherapy, with a mean age of 61.4 years, SD = 13.9 (5 women–38.5% and 8 men–61.5%).

The time after stroke for study participants was as follows: 5.2 years (SD = 2.7) in the dry land therapy group; 5.1 years (SD = 4.2) in the aquatic Ai Chi group; and 5.6 years (SD = 3.6) in the combined therapy group. The study was conducted between February and September 2018, during which the intervention was administrated and results were collected (pre-post and at the end of the intervention in each of the groups). All the participants completed all the sessions and complied with the proposed program. No adverse events were reported in relation to the interventions.

Table 1 shows the changes observed in the different variables under study in the three measurements made. In the experimental group, significant differences were found in the VAS and resilience variables (*p* < 0.001). A greater variation was observed in the groups who received aquatic interventions (Ai Chi and combined therapy). These changes were more significant in the post-intervention measurements.

Concerning the values obtained in the resilience scale, in the experimental group and aquatic therapy, significant differences were observed compared to the results obtained in the control group (dry land therapy), and these improvements were maintained one month after completing the treatment program. 

Table 2 displays the scores obtained on the SF-36 for each of the sections of the scale. We found significant differences (*p* < 0.001) in both the experimental and combined therapy groups, except in the areas of general health, vitality, and social function, where no perceptible differences in individuals were shown (*p* = 0.001). Conversely, in the group of people who received therapy on dry land, there were no significant differences in all the sections evaluated on the SF-36 scale. When analyzing the overall results of this scale, only the group that received therapy on dry land failed to show statistically significant difference after treatment. It is also important to note that in the groups that received aquatic therapy (Ai Chi and combined), positive outcomes were maintained one month after completing the proposed intervention.

**Figure 2:**
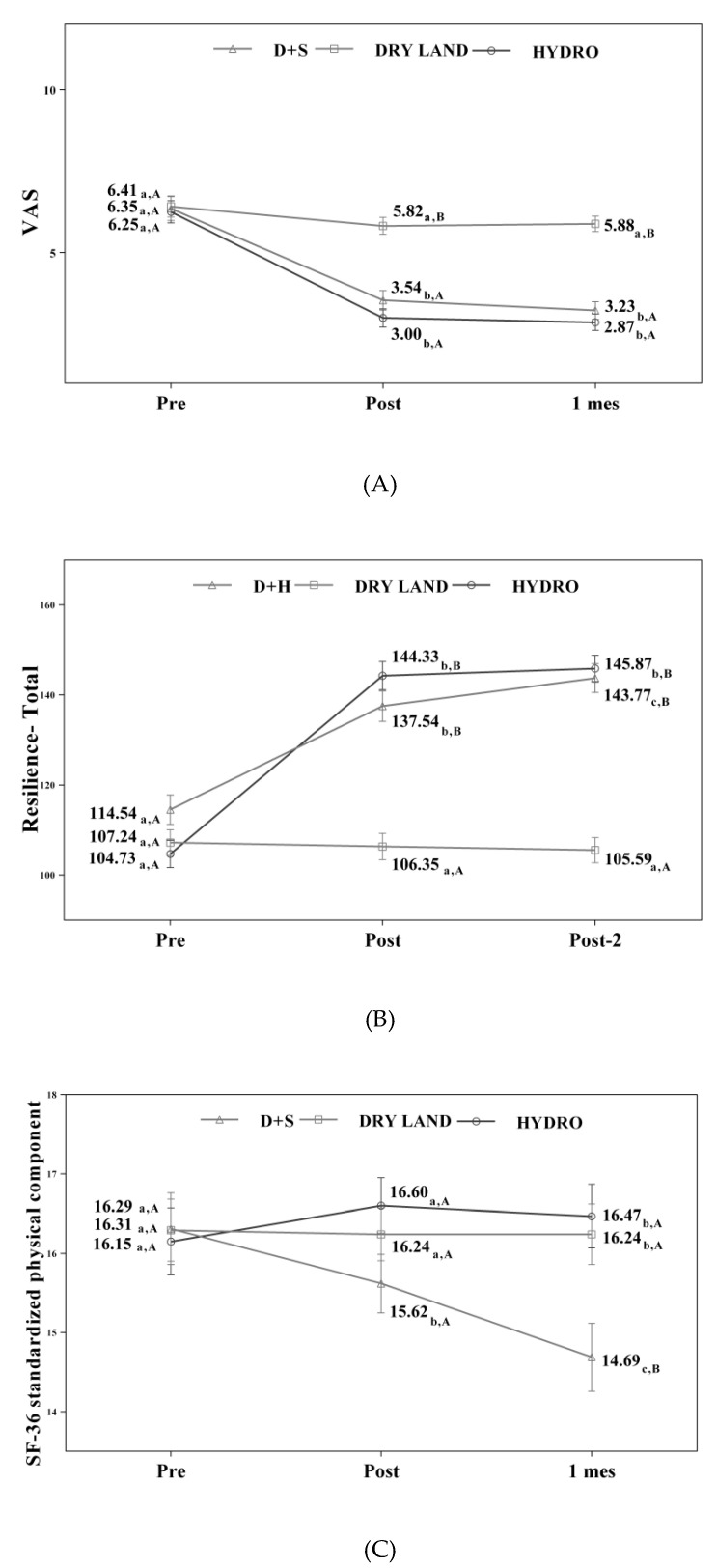
(**A**) Values of the VAS scale; (**B**) Values of the resilience scale; (**C**) Values of the SF-36 scale standardized physical component; D+S: combined therapy group.

Figure 2 displays changes observed in the values of each of the scales used in the assessment of the participating groups.

## 4. Discussion

This study investigated the effectiveness of a program of aquatic therapy, dry land physiotherapy and/or combined therapy on cognitive and emotional function in stroke patients. These findings show that the use of aquatic therapy in the treatment of stroke is beneficial for improving the quality of life and functionality of patients.

A systematic review carried out by Rafsten et al. [23] in 2018 described thirty-seven studies, comprising 13,756 patients, to examine the prevalence of anxiety, reporting an incidence of 29.3% of patients with stroke during the first year after the accident. Anxiety was particularly high during the hospitalization period (29.5%), rising to 36.7% in the first two weeks. The general estimate is that at least one in every three patients will develop some type of psychiatric disorder. These findings are comparable to a previous study [24]. Likewise, another study in Spain reported similar results, showing that highly functional stroke survivors also show a decline to their quality of life. One of the suggested reasons for this situation was the likely patient dissatisfaction with their inability to fully return to previous stroke levels [25].

Anxiety may be one of the predictors of depression [26,27]. Considering this statement, and the fact that anxiety after stroke is common, anxiety management may be useful to prevent depression in this type of patients. Thus, it is important to remember that psychological conditions significantly influence the quality of life of this population [28].

Regular physical exercise decreases anxiety symptoms [29]. This is related to the levels of b-endorphin and dopamine induced after physical activity, providing a calming effect on people who regularly practice sport. The study also showed that physical exercise in an aquatic environment improves the psychological state of the participants in the groups that received all or part of their therapy in the aquatic environment. Clinical trials involving low intensity aquatic exercises present significant results for the reduction of anxiety and antidepressant effects, corroborating the findings described by this study [29,30,31,32,33].

Anxiety and depression are characteristic symptoms of a person who has failed to respond appropriately to stressful life situations. Resilience is an individual’s ability to adapt, adequately and successfully, to a situation of acute stress, trauma, or chronic forms of adversity [34]. For this to occur, individuals must structure their adverse experience in a way that they are able to reconfigure their experiential elements, that is, by “reorganizing” their memory. This suggests that processes such as learning, representation, and contextual discrimination are consolidated at the level of memory and neuronal reorganization. This requires a highly plastic and multifunctional, motivational, emotional, and cognitive neuronal system. This type of integration is necessary for the subject to evaluate the setting (external stimuli, spatial and temporal parameters, location of the subject, internal stimuli, and even the possible threats related to the environment) and to select the most suitable strategy at that moment. The interactions of the hippocampus, ventral tegmental area (VTA), nucleus accumbens (NAc), and amygdala have been associated with representation, evaluation, and context modification [35,36,37]. Ultimately, the aquatic environment provides the patient with a wide range of external stimuli provided by the physical properties of the water, such as buoyancy, viscosity, and the need to adjust the response to changing environmental conditions, such as turbulence and depth. In addition to this, the task of having to learn a series of movements (19 positions and their corresponding transfers) induces neuroplasticity, a paramount process during a subject’s rehabilitation, in order to provide an adaptive response to adverse changes in the environment. Moreover, it is likely that resilient subjects may be able to reorganize traumatic memories thanks to the synaptic plasticity of the anatomical structures involved in this process [35,37]. These changes will allow them to adapt to adverse conditions, whereas, in this study, subjects who received the “dry land” physiotherapy program presented a much poorer resilience response compared with the other two groups.

Considering that motor recovery after stroke tends to reach a plateau phase after ten weeks, the initial improvement in cognitive function may follow a similar pattern [38], thus suggesting that an early specific and intensive intervention may be beneficial. By combining aerobic exercise with other training programs (in our case, exercises in an aquatic environment), cognitive function appears to improve, which should be considered when designing future performance protocols.

The motor disorders which, together with pain, are associated with impaired quality of life, can be reflected in the values obtained with the SF-36 scale. In this study, the values obtained after a dry land and/or aquatic environment approach revealed interesting results. All the items evaluated in the groups that received the aquatic environment intervention have shown significant improvements, more evident than those obtained in the group that received therapy on dry land. However, it is important to point out that three variables did not show such marked differences between the study groups: general health, vitality, and social function. This may be partly because in all three therapy options, the activity was carried out in a group format, which is an incentive for all participants to become involved in the proposed therapy. Thus, interpersonal relations (physiotherapists and patients) provide social support and an increased interest, as patients look towards their peers and see a potential relief from their personal situation. It is also important to emphasize that this positive change in their perceived quality of life is maintained in the aquatic therapy groups for at least one month after the end of the proposed sessions. 

Clearly, pharmacological therapy with antidepressants improves depression and anxiety levels for these patients [39,40]. However, medication may present certain contraindications at the motor, cognitive, and/or autonomic levels. For this reason, an aquatic therapy program proposed for this type of pathology may represent an effective treatment method that is free from side effects, as an adjuvant treatment to mitigate the physical and cognitive consequences of the stroke. The advantages of physical exercise in water include a greater variety of possible movements, the ability to perform easy and low-impact exercises in a pleasant and leisurely environment, along with the possibility of working in a group. All of this has a positive effect on this population, along with the water temperature [41]. This latter factor means that, on a therapeutic level, certain motor symptoms such as increased muscle tone and postural instability improve in this environment with these types of patients.

However, not all studies have shown positive results such as those presented here. An 18-month program of aquatic therapy among a group of stroke patients failed to find an advantage for the control of depression and anxiety [41]. According to the authors, these findings may have been due to the lack of adherence to the sessions on behalf of the selected sample. This was not the case with our sample, and therefore we can affirm that this conclusion may perhaps be extrapolated to other interventions preformed in the aquatic environment.

The main limitation of this study was the sample size. A larger sample would provide greater certainty regarding the findings obtained. The relatively short follow-up is an additional limitation, which should be considered in future related studies. Furthermore, these effects are only applicable to patients whose characteristics are similar to this sample. Therefore, future studies with larger sample sizes are necessary to confirm these findings.

## 5. Conclusions

In conclusion, the results of this study indicate that physical activity performed in the aquatic environment has positive effects on certain elements affecting the mood, pain, and quality of life of people who have suffered a stroke compared to treatment on dry land. Therefore, this treatment option should be considered when designing treatments protocols on behalf of neurologists and health professionals working in the field of neurorehabilitation, to ensure the necessary resources for its implementation.

## Figures and Tables

**Table 1:** Values obtained on the VAS and resilience scales.

	Pre	Post	1 Month	Time	Treatment*Time
	Mean (SD)	Mean (SD)	Mean (SD)	F(g.l.); *p*-Value (*eta*^2^)	F(g.l.); *p*-Value (*eta*^2^)
VAS Scale				F(1.3;56.5) = 158.28; *p* < 0.001 (0,790)	F(2.7;56.5) = 24.91; *p* < 0.001* (0.543)
H+D (13)	6.35 (1.0)	3.54 (0.8)	3.23 (0.9)		
Dry Land (17)	5.41 (1.5)	5.82 (1.1)	5.88 (1.0)		
AT (15)	6.25 (1.4)	3.00 (1.3)	2.87 (1.0)		
Personal Satisfaction resilience				F(1.2;52.3) = 67.77; *p* < 0.001 (0.617)	F(2.5;52.3) = 27.13; *p* < 0.001* (0.564)
H+D (13)	18.62 (3.1)	23.69 (3.2)	24.54 (3.8)		
Dry Land (17)	1665 (3.2)	16.59 (3.1)	16.24 (2.9)		
AT (15)	18.20 (2.6)	24.80 (3.4)	25.07 (3.5)		
Feeling Good on My Own resilience				F(1.4;58.5) = 77.50; *p* < 0.001 (0.649)	F(2.8;58.5) = 21.25; *p* < 0.01* (0.503)
H+D (13)	13.15 (2.8)	14.92 (3.3)	16.46 (3.2)		
Dry Land (17)	14.77 (2.4)	14.94 (2.2)	14.77 (2.1)		
AT (15)	14.33 (2.4)	19.67 (1.2)	20.27 (1.0)		
Self Confidence resilience				F(1.1;47.4) = 85.14; *p* < 0.001 (0.670)	F(2.3;47.4) = 25.09; *p* < 0.001* (0.544)
H+D (13)	29.62 (5.9)	37.54 (5.2)	38.85 (5.3)		
Dry Land (17)	29.82 (4.8)	29.71 (5.0)	29.47 (4.7)		
AT (15)	29.73 (5.1)	38.73 (3.4)	39.07 (3.2)		
Equanimity resilience				F(1.1;47.9) = 92.26; *p* < 0.001 (0.687)	F(2.3;47.9) = 58.82; *p* < 0.001* (0.737)
H+D (13)	15.15 (2.8)	22.54 (2.0)	22.92 (1.8)		
Dry Land (17)	14.65 (1.6)	14.53 (1.4)	14.35 (1.3)		
AT (15)	14.40 (2.1)	21.40 (2.6)	21.67 (2.4)		
Perseverance resilience				F(1.3;55.3) = 91.52; *p* < 0.001 (0.685)	F(2.6;55.3) = 35.92; *p* < 0.001* (0.631)
H+D (13)	31.50 (4.7)	38.85 (4.8)	41.00 (4.5)		
Dry Land (17)	31.35 (4.3)	30.59 (4.4)	30.18 (4.0)		
AT (15)	30.87 (5.3)	39.73 (4.7)	39.80 (4.6)		
Total resilience				F(1.2;51.7) = 167.48; *p* < 0.001 (0.799)	F(2.5;51.7) = 57.91; *p* < 0.001* (0.734)
H+D (13)	114.54 (13.9)	137.54 (14.7)	143.77 (15.2)		
DRY LAND (17)	107.24 (11,6)	106.35 (11.1)	105.59 (9.3)		
AT (15)	104.73 (9.8)	144.33 (10.8)	145.87 (9.8)		

Note: H+D: hydrotherapy + dry land; AT: aquatic therapy; SD: standard deviation; *eta*^2^: partial Eta squared (effect size); *: Greenhouse-Geisser correction; *p* < 0.001*: statistically significant difference.

**Table 2:** Values obtained on the SF-36.

	Pre	Post	1 Month	Time	Treatment*Time
	Mean (SD)	Mean (SD)	Mean (SD)	F(g.l.); *p*-Value (*eta*^2^)	F(g.l.); *p*-Value (*eta*^2^)
Physical Function				F(1.2;51.8) = 149.34; *p* < 0.001 (0.781)	F(2.5;51.8) = 36.90; *p* < 0.001* (0.637)
H+D (13)	16.92 (4.4)	22.08 (3.4)	23.46 (3.5)		
Dry Land (17)	17.06 (2.5)	17.24 (2.8)	17.24 (2.8)		
AT (15)	17.07 (3.5)	23.20 (2.5)	24.47 (2.4)		
Physical Role				F(1.3;53.5) = 87.19; *p* < 0.001 (0.675)	F(2.5;53.5) = 16.60; *p* < 0.001* (0.442)
H+D (13)	5.15 (1.4)	7.62 (1.0)	7.85 (0.6)		
Dry Land (17)	5.24 (1.6)	5.59 (1.4)	5.59 (1.4)		
AT (15)	5.07 (1.2)	7.60 (0.6)	7.93 (0.3)		
Body Pain				F(1.3;53.8) = 134.65; *p* < 0.001 (0.762)	F(2.6;53.8) = 36.34; *p* < 0.001* (0.634)
H+D (13)	6.93 (2.6)	3.39 (1.2)	2.77 (0.7)		
Dry Land (17)	6.94 (1.1)	6.94 (1.2)	6.94 (1.2)		
AT (15)	7.00 (1.3)	3.67 (0.7)	2.93 (1.0)		
General Health				F(1.7;71.6) = 8.00; *p* = 0.001 (0.160)	F(3.4;71,6) = 3.13; *p* = 0.026 (0.130)
H+D (13)	16.31 (2.1)	15.62 (1.7)	14.69 (2.1)		
Dry Land (17)	16.29 (1.3)	16.24 (1.4)	16.24 (1.4)		
AT (15)	16.15 (1.5)	16.60 (0.8)	16.47 (1.1)		
Vitality				F(1.2;48.9) = 1.72; *p* = 0.196 (0.039)	F(2.3;48.9) = 3.69; *p* = 0.026 (0.149)
H+D (13)	16.23 (1.3)	15.31 (1.3)	15.77 (1.6)		
Dry Land (17)	16.29 (2.2)	16.65 (2.0)	16.65 (2.0)		
AT (15)	16.21 (2.2)	15.47 (0.6)	15.63 (0.9)		
Social Function				F(1.3;56.1) = 5.25; *p* = 0.017 (0.111)	F(2.7;56.1) = 1.75; *p* = 0.172 (0.077)
H+D (13)	5.71 (0.9)	5.85 (0.8)	6.15 (0.7)		
Dry Land (17)	5.69 (1.2)	5.65 (1.2)	5.65 (1.2)		
AT (15)	5.72 (1.2)	6.20 (0.7)	6.13 (0.6)		
Emotional Role				F(1.3;53.3) = 28.50; *p* < 0.001 (0.404)	F(2.5;53.3) = 10.94; *p* < 0.001* (0.343)
H+D (13)	4.46 (1.2)	5.31 (1.1)	5.62 (0.9)		
Dry Land (17)	4.65 (1.0)	4.53 (0.9)	4.53 (0.9)		
AT (15)	4.67 (1.0)	5.93 (0.3)	6.00 (0.0)		
Mental Health				F(1.4;58.4) = 41.05; *p* < 0.001 (0.494)	F(2.8;58.4) = 8.83; *p* < 0.001* (0.296)
H+D (13)	20.00 (2.6)	20.77 (1.7)	21.69 (1.7)		
Dry Land (17)	20.35 (1.1)	20.53 (1.1)	20.53 (1.1)		
AT (15)	20.40 (1.5)	22.20 (1.1)	22.73 (0.9)		

Note: H+D: hydrotherapy + dry land; AT: aquatic therapy; SD: standard deviation; *eta*^2^: partial Eta squared (effect size); *: Greenhouse-Geisser correction; *p* < 0.001*: statistically significant difference.
